# Expected Evolution of COVID-19 Epidemic in France for Several Combinations of Vaccination Strategies and Barrier Measures

**DOI:** 10.3390/vaccines9121462

**Published:** 2021-12-10

**Authors:** Simon Pageaud, Catherine Pothier, Christophe Rigotti, Anne Eyraud-Loisel, Jean-Pierre Bertoglio, Alexis Bienvenüe, Nicolas Leboisne, Nicolas Ponthus, Romain Gauchon, François Gueyffier, Philippe Vanhems, Jean Iwaz, Stéphane Loisel, Pascal Roy, on behalf of the Group CovDyn (Covid Dynamics)

**Affiliations:** 1 Université de Lyon, F-69000 Lyon, France; simon.pageaud@gmail.com (S.P.); francois.gueyffier@univ-lyon1.fr (F.G.); jean.iwaz@chu-lyon.fr (J.I.); 2 Université Claude Bernard Lyon 1, F-69100 Villeurbanne, France; 3 CNRS UMR 5558, Laboratoire de Biométrie et Biologie Évolutive, F-69100 Villeurbanne, France; 4 Service de Biostatistique-Bioinformatique, Pôle Santé Publique, Hospices Civils de Lyon, F-69003 Lyon, France; 5 Laboratoire de Sciences Actuarielle et Financière (LSAF), Institut de Science Financière et d’Assurances (ISFA), Université Claude Bernard Lyon 1, F-69007 Lyon, France; anne.loisel@univ-lyon1.fr (A.E.-L.); alexis.bienvenue@univ-lyon1.fr (A.B.); nicolas.leboisne@univ-lyon1.fr (N.L.); romain.gauchon@laposte.net (R.G.); stephane.loisel@univ-lyon1.fr (S.L.); 6 Fondation du Risque, Groupe Louis Bachelier, F-75002 Paris, France; 7 CNRS UMR 5205, Laboratoire d’InfoRmatique en Image et Systèmes d’Information (LIRIS), F-69621 Villeurbanne, France; catherine.pothier@insa-lyon.fr (C.P.); christophe.rigotti@insa-lyon.fr (C.R.); 8 Institut National des Sciences Appliquées de Lyon (INSA), F-69621 Villeurbanne, France; 9 INRIA Grenoble-Rhône-Alpes, F-38334 Montbonnot, France; 10 CNRS UMR 5509, Laboratoire de Mécanique des Fluides et d’Acoustique (LMFA), F-69130 Ecully, France; jean-pierre.bertoglio@ec-lyon.fr; 11 École Centrale de Lyon, F-69130 Lyon, France; nicolas.ponthus@gmx.fr; 12 CNRS UMR 5513, Laboratoire de Tribologie et Dynamique des Systèmes (LTDS), F-69130 Ecully, France; 13 École Nationale des Travaux Publics de l’État (ENTPE), F-69120 Vaulx-en-Velin, France; 14 Service d’Hygiène, Épidémiologie, Infectiovigilance et Prévention, Hôpital Edouard Herriot, Hospices Civils de Lyon, F-69003 Lyon, France; philippe.vanhems@chu-lyon.fr; 15 Centre International de Recherche en Infectiologie (CIRI: Inserm U1111, CNRS UMR 5308, École Nationale Supérieure de Lyon), F-69007 Lyon, France

**Keywords:** vaccination, COVID-19, agent-based model, decision support techniques

## Abstract

The outbreak of the SARS-CoV-2 virus, enhanced by rapid spreads of variants, has caused a major international health crisis, with serious public health and economic consequences. An agent-based model was designed to simulate the evolution of the epidemic in France over 2021 and the first six months of 2022. The study compares the efficiencies of four theoretical vaccination campaigns (over 6, 9, 12, and 18 months), combined with various non-pharmaceutical interventions. In France, with the emergence of the Alpha variant, without vaccination and despite strict barrier measures, more than 600,000 deaths would be observed. An efficient vaccination campaign (i.e., total coverage of the French population) over six months would divide the death toll by 10. A vaccination campaign of 12, instead of 6, months would slightly increase the disease-related mortality (+6%) but require a 77% increase in ICU bed–days. A campaign over 18 months would increase the disease-related mortality by 17% and require a 244% increase in ICU bed–days. Thus, it seems mandatory to vaccinate the highest possible percentage of the population within 12, or better yet, 9 months. The race against the epidemic and virus variants is really a matter of vaccination strategy.

## 1. Introduction

The outbreak of SARS-CoV-2 virus is causing major national, European, and international health crises, with serious public health and economic consequences. The COVID-19 epidemic started in China, in late autumn 2019, and, on 31 December [1], the WHO China office received reports on severe pneumonia cases in the city of Wuhan (Hubei Province). On 14 February 2021, more than 100 million cases and 2.4 million officially recognized deaths were recorded worldwide, of which there were 34 million cases and 760 thousand deaths in Europe and 3.4 million cases and 81.6 thousand deaths in France. In most countries, the dynamics of the epidemic is continuously assessed through monitoring the number of hospital admissions, occupancy rate of intensive care units (ICUs), and cumulative number of confirmed COVID-19-related deaths.

In France, in 2020, two lockdown periods were imposed to avoid saturation of ICUs that would increase mortality. The first period (17 March to 11 May 2020) aimed at limiting the severity of the first wave. On 8 April 2020, the maximum number of daily hospital deaths (a 7-day moving average) was 532 and, between 6 June and 16 September 2020, less than 30 daily hospital deaths being observed to form a plateau. At the beginning and end of the second lockdown (30 October and 15 December 2020, respectively), the numbers of daily hospital deaths were 222 and 276, respectively, and a maximum of 419 deaths was reached on 19 November. Nevertheless, the resulting epidemic situation was less favorable after the second lockdown; it showed a residual plateau with 240 to 340 daily hospital deaths.

On 14 December 2020, the Alpha variant (lineage B.1.1.7) was identified in the United Kingdom [2], whereas, in France, the first case was identified by the end of December. The World Health Organisation designated that variant as a ‘variant of concern’ (VOC), which would require new appropriate public health actions. In France, the Alpha variant was deemed responsible for 3.3% of diagnosed cases on 8 January 2021, 14% on 27 January, and 36% on 16 February. Preliminary studies suggested that it increased transmissibility (infectiousness) but not disease severity. The prevalence of this variant in France is available from the public data of Santé Publique France (https://www.santepubliquefrance.fr/, accessed on 15 October 2020). Lineage B.1.672 of SARS-CoV-2 was first detected in December 2020 in India [3], but it appeared only months later in France. The Delta variant (lineage B.1.617.2) was labeled as a VOC on 11 May 2021; on that date, in France, it was deemed responsible for 1% of diagnosed cases.

Vaccines against COVID-19 have been rapidly developed to limit the spread of the epidemic through herd immunity after reaching a high vaccination coverage. Their efficacy against the severe forms of the disease, caused by the historical strain, was estimated to lie between 75% [4] and 94% [5,6]. By the time of those vaccine releases, their efficacy in preventing disease transmission was not known.

In France, protective measures have been constantly adapted to the dynamics of the epidemic. Between September 2020 and January 2021, three periods with different levels of non-pharmaceutical interventions (NPIs) took place. The first period (September 2020–October 2020) corresponded to moderate-NPIs, such as mask wearing and social distancing, but restaurants and leisure facilities remained open. The second period (November 2020) was characterized by extended-NPIs, which consisted of very strict measures, such as lockdown during weekends or full lockdown, as well as closing of schools, universities, and shopping facilities. The third period (December 2020–January 2021) was characterized by intensive-NPIs (intermediate between moderate-NPIs and extended-NPIs), combining several strict protective measures, such as overnight curfew, social distancing, mask-wearing, and increased distancing at work, as well as the closing of bars, restaurants, leisure facilities, etc.

This study aimed to predict the course of the COVID-19 epidemic over an 18-month period, starting on 1 January 2021, by applying several vaccination campaign and barrier measure combinations and taking into account the increasing proportion of the Alpha variant. It was based on a previously published scientific report on a non-calibrated theoretical version [7]. (Note: the proposed displayed results were obtained before the emergence of the Delta variant).

## 2. Results

### 2.1. Dynamics of the Epidemic with the Historical Strain without Vaccination

Table 1 presents the results obtained by restricting the simulations to the historical strain without vaccination and setting the prevalence of asymptomatic cases at 20%.

**Figure 1:**
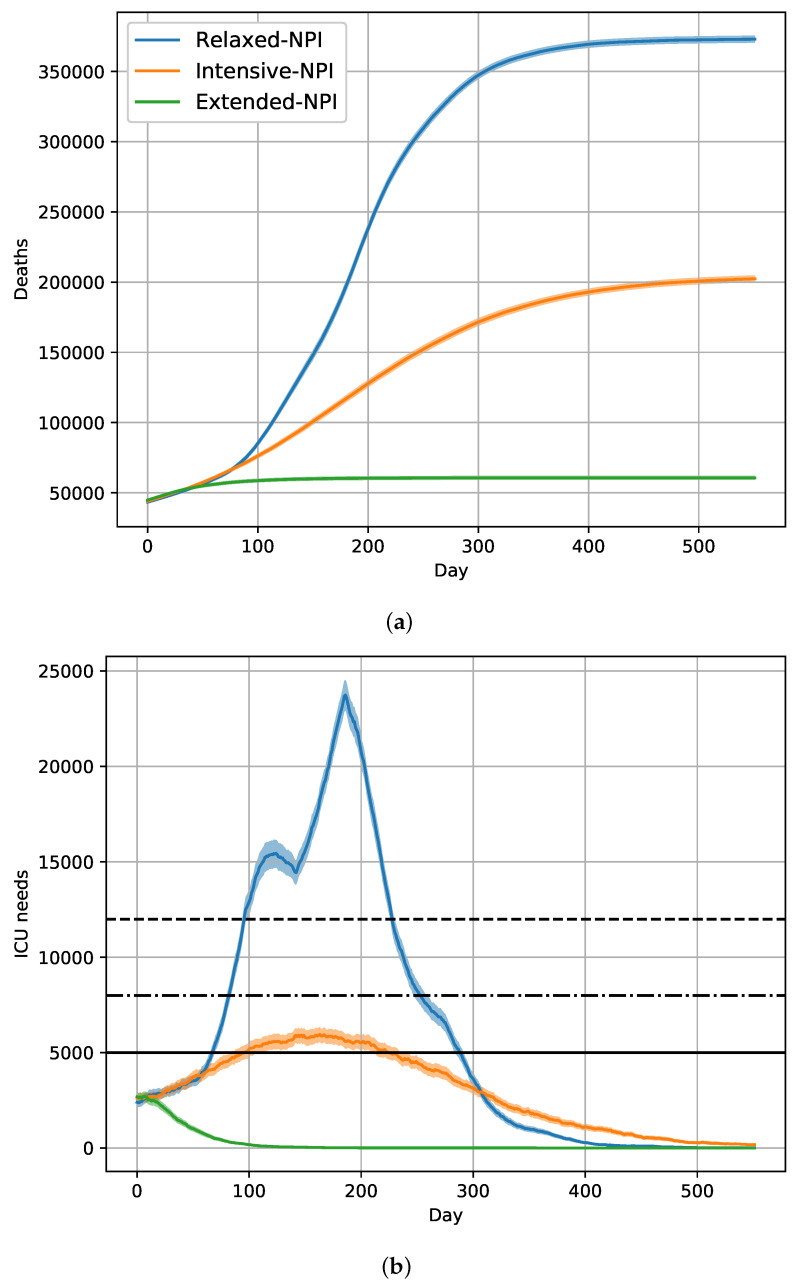
Expected evolution of the COVID-19 epidemic in France for the historical strain, without vaccination, for extended-NPIs, intensive-NPIs, and relaxed-NPIs, as well as for 20% rate of asymptomatic subjects. Day 0 is 1 January 2021; (**a**) cumulative number of deaths. (**b**) Prevalence of COVID-19 patients needing intense care units. The horizontal black lines represent ICU capacities of 5000, 8000, and 12,000 beds.

After 18 months of relaxed-NPIs (systematic alternation of periods of 45 days between intensive-NPIs and moderate-NPIs), the simulated number of removed individuals was 26.4 millions, and the number of deaths was 373 thousands. In this scenario, the cumulative distribution of deaths plateaued after 500 days (Figure 1a). A maximum daily requirement of 23,732 ICU beds were needed on day 186 (Figure 1b). A slightly delayed effect of changes between intensive and moderate protection measures on ICU needs was observed under relaxed-NPIs, and the control of the epidemic was obtained after 500 days.

After 18 months of intensive-NPIs, the simulated number of removed individuals was much lower (around 16.7 millions), with 202.4 thousand deaths. The cumulative distribution of deaths was still increasing after 500 days (Figure 1a). A maximum daily requirement of 5960 ICU beds were needed on day 163 (Figure 1b). A slow decrease was then observed, indicating a control of the epidemic after 500 days of intensive-NPIs.

After 18 months of extended-NPIs, the simulated number of removed individuals was 7.9 millions, with 60.8 thousand deaths. The cumulative distribution of deaths reached a plateau after 150 days (Figure 1a). A maximum daily requirement of 2700 ICU beds were needed on day 6 (Figure 1b). A rapid decrease was observed, indicating a control of the epidemic by day 150 under extended-NPIs.

Figure 1 shows clearly distinct changes in death (Figure 1a) and ICU beds occupation (Figure 1b), when relaxed-NPIs, intensive-NPIs, and extended-NPIs were applied, respectively.

### 2.2. Dynamics of the Epidemic with the Introduction of the Alpha Variant without Vaccination

**Figure 2:**
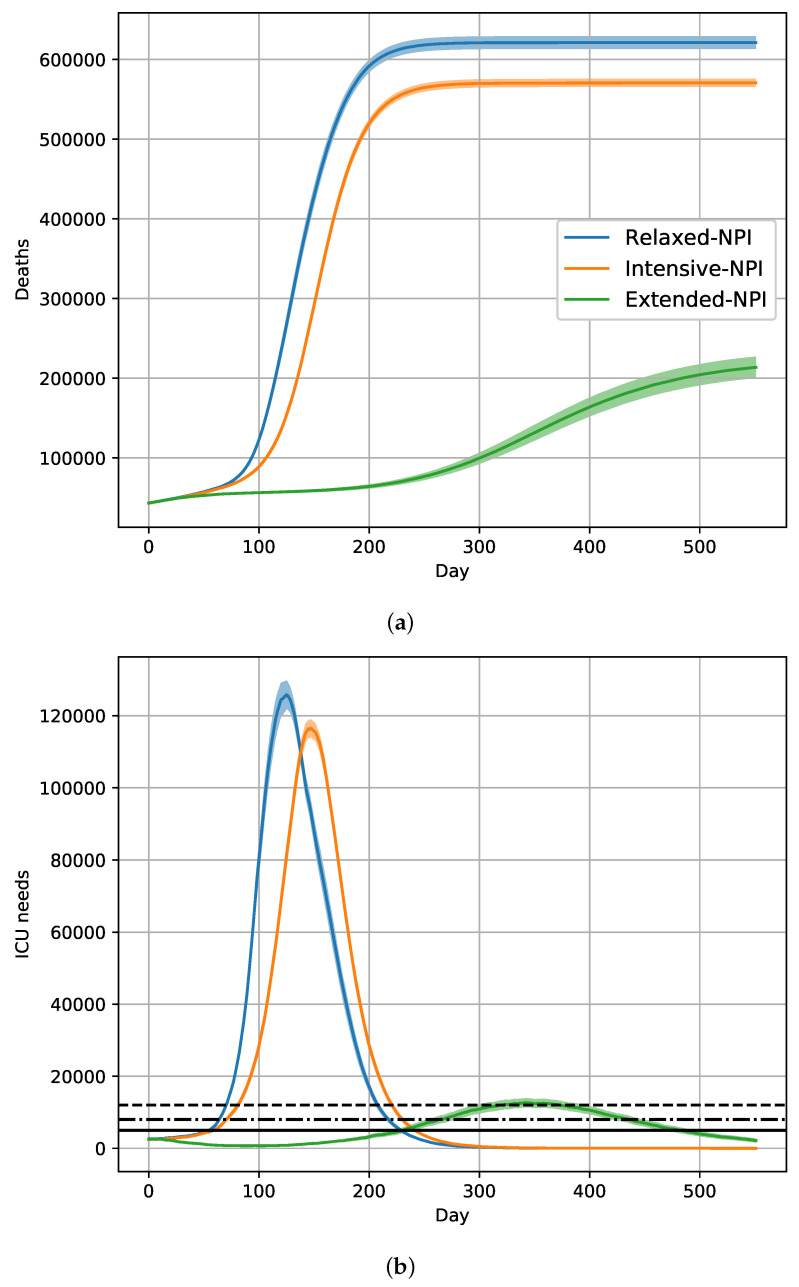
Expected evolution of the COVID-19 epidemic in France with the historical strain and Alpha variant, without vaccination, for extended-NPIs, intensive-NPIs, and relaxed-NPIs, as well as for 20% rate of asymptomatic subjects. Day 0 is 1 January 2021; (**a**) cumulative number of deaths. (**b**) Prevalence of COVID-19 patients needing Intense Care Units. The horizontal black lines represent an ICU capacity of 5000, 8000, and 12,000 beds.

Table 2 presents the results obtained in a situation of competition between the historical strain and the more infectious Alpha variant in the absence of vaccination. With relaxed-NPIs, the simulated number of removed individuals was around 48.3 millions, with 621.3 thousand deaths. The cumulative distribution of deaths reached a plateau at day 300 (Figure 2a). A maximum daily requirement of 125,824 ICU beds were needed on day 125. A huge single wave occurred, without possible distinction between intensive-NPIs and moderate-NPIs periods of 45 days. With relaxed-NPIs, a control of the epidemic occurred at day 350 (Figure 2b).

With intensive-NPIs, the simulated number of removed individuals was still important (around 45.5 millions), with 570.6 thousand deaths. The cumulative distribution of deaths reached a plateau after 300 days (Figure 2a). A maximum daily requirement of 116,548 ICU beds was needed on day 147. Despite continuous intensive-NPIs, a huge single wave was observed, and the control of the epidemic occurred at day 350 (Figure 2b).

With extended-NPIs, the simulated number of removed individuals was 20.6 millions, with 213.7 thousand deaths. The cumulative distribution of deaths still increased at 500 days (Figure 2a). A maximum daily requirement of 12,650 ICU beds was needed on day 343. In comparison to the wave simulated under intensive-NPIs, the wave under extended-NPIs was delayed, and the control of the epidemic did not occur at day 500 (Figure 2b).

With relaxed- and intensive-NPIs, close simulated results were obtained, in terms of mortality and ICU overload. Only extended-NPIs seemed able to partially limit the number of deaths without ensuring herd immunity (Figure 2).

### 2.3. Effects of a 6-Month Vaccination Campaign with Intensive-NPIs

**Figure 3:**
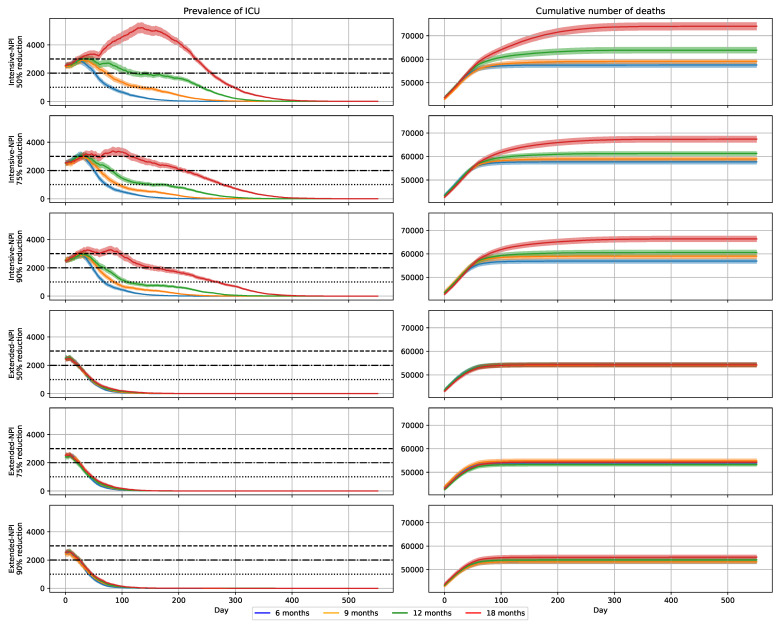
Expected evolution of the COVID-19 epidemic in France for the historical strain and Alpha variant, for different durations of vaccination campaigns and reductions of virus transmission of 50%, 75%, and 90%. Each row displays the prevalence of COVID-19 patients needing intensive care and cumulative number of deaths at hospital for 20% rate of asymptomatic subjects. Day 0 is 1 January 2021.

Under the assumption of a 90% reduction in virus transmission, due to the vaccine, the number of individuals aged 10 years and older removed by the disease (8.6 millions) was divided by 5, and the number of deaths (56.9 thousands) was divided by 10 (Table 3). The number of deaths reached a plateau at day 200 (Figure 3).

The ICUs never reached saturation (Figure 3), although ICU bed requirements exceeded 1000 beds for 69 days and 2000 beds for 48 days. That need never exceeded 3000 beds. A total of 192,728 ICU bed–days were required, with a maximum daily requirement of 2926 beds on day 30.

In comparison with vaccination of individuals aged 10 years and older, vaccination of people of all ages resulted in an additional 0.8% reduction in the number of deaths.

### 2.4. Effects of a 9-Month Vaccination Campaign with Intensive-NPIs

Under the assumption of a 90% reduction in virus transmission, due to the vaccine, the number of individuals aged 10 years and older removed by the disease (9.4 millions) and number of deaths (59.0 thousands) were quite similar to those obtained with a 6-month vaccination campaign (Table 3). The number of deaths reached a plateau at day 250 (Figure 3).

The pressure on ICUs was increasing (Figure 3); 253,715 ICU bed–days were required.

### 2.5. Effects of a 12-Month Vaccination Campaign with Intensive-NPIs

Under the assumption of a 90% reduction in virus transmission, due to the vaccine, the number of individuals aged 10 years and older removed by the disease (10.7 millions) and the number of deaths (60.6 thousands) were slightly higher than those obtained with 6-month and 9-month vaccination campaigns (Table 3). The number of deaths reached a plateau at day 300 (Figure 3).

The pressure on ICUs was still increasing (Figure 3); 341,083 ICU bed–days were required.

### 2.6. Effects of a 18-Month Vaccination Campaign with Intensive-NPIs

Under the assumption of a 90% reduction in virus transmission due to the vaccine, the number of individuals aged 10 years and older removed by the disease (14.2 millions) and the number of deaths (66.4 thousands) were, respectively, 32% and 10% higher than those obtained with a 12-month vaccination campaigns (Table 3). The number of deaths reached a plateau at day 400 (Figure 3).

The pressure on ICUs was much higher (Figure 3); 662,125 ICU bed–days were required.

### 2.7. Impact of Transmission Reduction on Vaccination Campaign Efficacy

With a vaccine less able to reduce viral transmission, the negative impacts of extending the vaccination campaign on mortality, and on the need for ICU beds, increased (Table 4 and Table 5).

With a transmission reduction of 90%, extending the vaccination campaign from 6 to 18 months increased the number of deaths from 56.9 to 66.4 thousands (+17%), and the number of ICU bed–days needed from 192,728 to 662,125 (+244%) (Table 3). With a transmission reduction of 50%, the number of deaths increased from 57.5 to 74.0 thousands (+29%) and the number of ICU bed–days from 216,751 to 1,106,397 (+410%) (Table 5).

### 2.8. Impact of Extended-NPIs on Vaccination Campaign Efficacy

With extended-NPIs, the impacts of the probability of viral transmission and duration of the vaccination campaign on mortality and ICU pressure were much smaller than with intensive-NPIs (Figure 3).

### 2.9. Reduction in Disease Transmission by NPIs and Relative Contagiousness of the Alpha Variant

Compared to moderate-NPIs, the estimated relative reductions in disease transmission were 0.783 for intensive-NPIs and 0.534 for extensive-NPIs ($\beta_{2,\mathrm{Intensive}}$ and $\beta_{2,\mathrm{Extended}}$, Table 6). With the historical strain as reference, the Alpha variant was estimated to be 1.572 times more infectious ($\beta_{3,\mathrm{Alpha}}$, Table 6).

The estimated proportion of removed individuals was 11% on 1 January 2021, and the estimated prevalence of the Alpha variant represented 13.2% of SARS-CoV-2 strains on 27 January 2021 and 38.4% on 16 February 2021.

## 3. Discussion

The modelling proposed in this study provides objective and original estimates of the effects of NPIs at the population level. With estimated reductions in the probability of COVID-19 transmission of 25% ($\beta_{2,\mathrm{Intensive}}=0.783$) and 50% ($\beta_{2,\mathrm{Extended}}=0.534$), intensive-NPIs and extended-NPIs have slowed the dynamics of the COVID-19 epidemic. The first part of this work simulated the spread of the epidemic in the absence of vaccination. With the historical strain alone, adopting relaxed-NPIs would have led to the extinction of the epidemic in about 400 days but at the cost of a very high mortality (about 372 thousand deaths) and a serious ICU overcrowding. Adopting extended-NPIs would have led to much less deaths (about 61 thousands), without ICU overloading. Intensive-NPIs would have led to intermediate results.

The estimated relative infectiousness of the Alpha variant ($\beta_{3,\mathrm{Alpha}}=1.572$) was in the range of the literature estimates [8]. The estimated prevalence values of the Alpha variant on 27 January 2021 (13.2%) and 16 February 2021 (38.4%) were close to the observed values on those dates (i.e., 14% and 36%, respectively). These results support the proposed multiplicative approach (see Methods, Equation (1)).

Strikingly, in the absence of vaccination, as soon as the variant strain was included in the simulations, extended-NPIs had to be imposed to avoid large increases in the number of deaths and important ICU overload as intensive-NPIs became ineffective. Extended-NPIs should be maintained, as long as herd immunity is achieved. The population would not be protected against a new epidemic wave if these protective measures were removed. These conclusions are in agreement with the recommendations of Bosetti et al. [9] that reinforced NPIs should be applied to more contagious variants to achieve an epidemic control, similar to that obtained by applying less stringent measures to the historical virus strain.

Within the current context, vaccination seems to be the sole solution to stop the epidemic; thus, it is important to vaccinate the whole population as soon as possible. Reaching herd immunity through vaccination is mandatory to avoid the resumption of the epidemic after NPIs removal. With intensive-NPIs and a vaccine reduction of virus transmission of 90%, extending the vaccination campaign from 6 to 12 months, would have a relatively moderate impact on the disease-specific mortality (increase by 6%) but a high impact on the need for ICU bed–days (+77%). However, maintaining intensive-NPIs would have deleterious health consequences, such as delayed surgical management of benign diseases, inadequate follow-up of chronic diseases, and disruptions of health prevention programs. Besides, intensive-NPIs have deleterious impacts on education, research, economy, etc. Thus, extending the vaccination campaign to 12 months would lead to significant pressure on ICUs and the entire health system, as well as to social impacts from maintaining intensive-NPIs during that period. A substantial increase in disease-related mortality of 17% would be seen if the vaccination campaign were extended over 18 months, with a strong need for ICU bed–days (+244%). Moreover, maintaining intensive-NPIs for 18 months seems unrealistic for social and economic reasons. All those elements are in favour of the fastest possible mass vaccination, providing vaccine availability and population compliance.

Despite a significant acceleration of the current French vaccination campaign, the objective of vaccinating the entire French population (67 millions) in 6, or even 9, months has definitely not been achieved. In France, the vaccination campaign started on 11 January 2021. By 1 April 2021, 3.1 million people were fully vaccinated and 9.0 millions had received a first dose and, by 1 June 2021, 12.5 million people were fully vaccinated (16% of the population) and 26.4 millions had received a first dose. On 1 June 2021, the cumulative number of observed hospital deaths was 84 thousands, whereas 65 thousand deaths were predicted by the worst-case scenario (Table 5). Two main reasons explain this difference. The first is the vaccination coverage rate among the elderly. In simulated vaccination campaigns, all people aged 75 years or older received a first dose of vaccine on day 46 of 2021 (15 February) for the 18-month vaccination campaigns, and earlier for shorter campaigns. In contrast, by 15 February 2021, only 22.2% of individuals aged 75 years or older had actually received a first dose of vaccine. The second reason concerns NPIs. A relaxation of NPIs was seen in France during the first half of 2021: the curfew starting at 6 PM, decided in January, was postponed to 7 PM in March, 9 PM in May, and 11 PM in June; the isolation of travelers entering the territory was eased during a part of that period; and some business activities reopened in February, March, and May. An important additional argument in favour of limiting the duration of the vaccination campaign is the difficulty of maintaining acceptance of appropriate NPIs over a long period.

Another argument in favour of rapid and extensive routine vaccination is the race against the emergence of new VOC that is directly related to the number of infected individuals. Within this context, our opinion is that vaccinating the entire population may be necessary after ensuring the safety and efficacy of vaccination of children (see NCT04796896 and NCT04816643 trials on clinicaltrials.gov).

The present study makes the strong hypothesis of a 100% acceptance level of vaccination. Virus extinction will happen if the population is widely vaccinated. This means that more efforts should be made, in terms of communication and vaccination strategy. It would be interesting to tackle the issue of vaccine acceptance by measuring the impacts of partial acceptance levels on the course of the epidemic. Another modelling choice was ignoring the duration of vaccine protection. This might be the object of future investigations.

The model presented in this paper has been calibrated on the historical strain, variants with contagiousness similar to that of the Alpha variant, virulence similar to that of the historical strain, and the absence of vaccine resistance. Once sufficient data will be available to estimate a realistic $\beta_{3}$ for each variant, this model will allow comparing the effects of various vaccination strategies on mixtures of virus strains. It will also be possible to incorporate a variant-specific vaccine resistance parameter into the model, as soon as meaningful estimates of that parameter will be available. An increase in the virus virulence may be handled by changing the model transition probabilities (see [10]).

The results presented and discussed here do not take the Delta variant into account because the model was designed and developed before the emergence of that variant. The study was, thus, focused on the effect of the Alpha variant and the model calibration used Alpha-variant-specific data. Therefore, the results obviously under-estimate the real impact of the whole range of existing variants. The model used is sufficiently flexible to be adapted to measure the impacts of the Delta variant on the vaccination strategies as soon as sufficient data will be available. This is a perspective for a future work, that would also integrate scenarios about future more dangerous and/or contagious variants.

One advantage of the modelling presented in this paper is that it takes into account the particularity of any vaccination strategy. If it appears that it will not be possible to vaccinate all the population within one year, alternative solutions will have to be examined. This work did not consider the logistics details of the vaccination strategies; nevertheless, the model flexibility allows including those details. In addition, future works may tackle the delay between the two vaccine doses or the pace of vaccination through increasing the number of vaccination centers, vaccinating personnel, or available vaccine supplies.

The present study considered the side effects of anti-COVID-19 vaccines to be negligible in comparison with the expected benefit at the population level (control of the epidemic through herd immunity). In fact, the rare serious side effects can be taken into account by considering the benefit-risk balance at an individual level, especially when the risk of severe COVID-19 is very low (e.g., in young children). In a safety analysis of BNT162b2, the mRNA COVID-19 vaccine in individuals aged 16 or older, the estimated excess of myocarditis associated with the vaccine was 2.7 events per 100,000 vaccinations (95% CI: 1.0 to 4.6), whereas the estimated excess of myocarditis associated with COVID-19 disease was 11.0 events per 100,000 cases (95% CI: 5.6 to 15.8) [11]. In a cohort of individuals aged 18–65 years, vaccinated with the ChAdOx1-S, the estimated excess of venous thromboembolic events was 11 events per 100,000 vaccinations (95% CI: 5.6 to 17.0), of which an excess of cerebral venous thrombosis of 2.5 events per 100,000 vaccinations (95% CI: 0.9 to 5.2) [12]. The reporting of those rare vaccine side effects influenced the French vaccination campaign by increasing the use of the BNT162b2 mRNA COVID-19 vaccine. Underestimating the vaccination benefit–risk balance might have a negative impact on the acceptance of a vaccine and even vaccination.

Other limitations concern taking into account the details of the transmission and the severity of the disease. In particular, the model did not consider the living conditions (e.g., residence in cities vs. villages, holidays, etc.) that can influence the number of contacts and the application of barrier measures; in addition, it did not consider the prevalence of comorbidities [13]. Fortunately, the agent-based model used in this study simulated encounters and transmissions between individuals; thus, it may be extended to include any selection of individual characteristics. Furthermore, as the simulation can be run with a large number of individuals (more than a million), it may represent various comorbidities, living, habits, or other conditions within a given population. Such improvements of the core model may be used to investigate the potential effects of targeted public health decisions.

## 4. Methods

Section 4.1 presents the agent-based model and Section 4.2 shows the simulated vaccination scenarios obtained from that model. The model calibration is presented Section 4.3.

### 4.1. The Agent-Based Model

**Figure 4:**
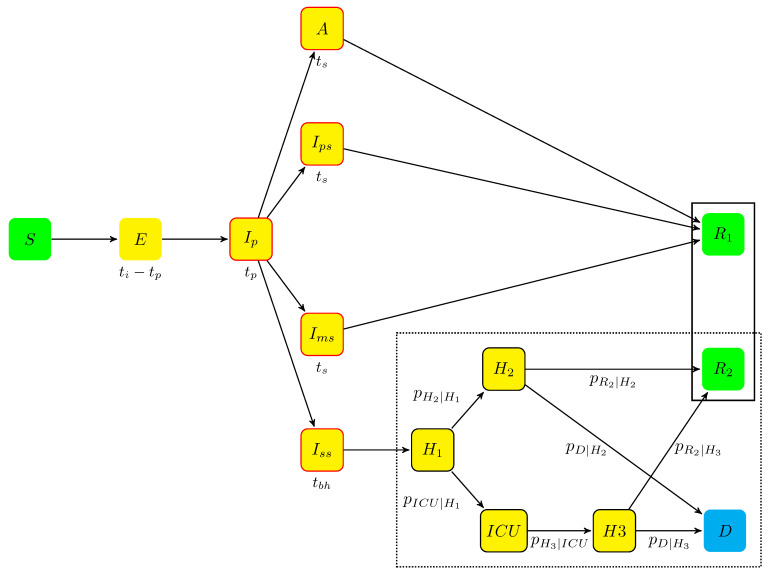
The states and their connections in the model. The left-hand part of the model represents the disease spreading with the average sojourn time beneath each compartment. The right-hand part of the model (bottom-right dotted frame) represents the hospitalized cases and their outcomes. It is composed of states $H_{1}$, $H_{2}$, $H_{3}$, ICU, $R_{2}$, and *D*. Each arrow is labeled with the individual daily transition probability between two states.

The agent-based model (ABM) allowed simulating individual trajectories between specific states of disease occurrence and evolution. This model (Figure 4) extended the set of compartments initially proposed by Di Domenico et al. [14] to an ordinary differential equation (ODE) model. The ABM included two parts. The left-hand side of the model, which corresponds to ‘disease spreading’, reproduced the compartment pattern of Di Domenico et al. [14]. The right-hand part (the dotted frame in Figure 4) describes the ‘outcome of hospitalized cases’ and includes specific states to account explicitly for hospitalization, before and after ICU periods. Each state of the model was divided into nine parts for the nine age groups, as described by Gauchon et al. [15] (only three age groups were considered in the model of Di Domenico et al. [14]). The flexibility of the ABM allowed incorporating vaccination, variant occurrence, and types of NPI measures (three aspects that were not considered by Di Domenico et al. [14]). In the ABM model, the transition probabilities—that are similar to those of a multi-state Markov model—were applied at individual levels. The states and trajectories are summarized in Figure 4; the parameters estimated or extracted from the literature are shown in Table 6 and Table 7.

#### 4.1.1. The Model States

The model is a stochastic ABM, with a discrete time step of one day. The states used in the model are the following:

- Individuals who were not infected by the virus begin in state *S* (susceptible);
- The incubation period includes two states:
  - when infected (see next section), an individual moves from state *S* to state *E* (exposed), which contains infected individuals who did not develop symptoms yet and are not contagious. The mean sojourn time in *E* was $\left(t_{i}-t_{p}\right)$, $t_{i}$ being the incubation period and $t_{p}$ the duration of the prodromal state (see next item);
  - when contagious, an individual is transferred from state *E* to state $I_{p}$ (prodromal phase, which is the short phase that follows contamination without symptoms but with possible non-specific prodromes). After an average stay $t_{p}$, the individuals moved to one of the four states *A*, $I_{ps}$, $I_{ms}$, and $I_{ss}$, defined hereafter.
- *A* (asymptomatic state, with probability $p_{a}$): individuals who completed the incubation period, became infectious, but without disease symptoms. The mean sojourn time in *A* was $t_{s}$;
- The symptomatic infectious period includes three states for individuals developing symptoms, (with probability $1-p_{a}$):
  - $I_{ps}$ (paucisymptomatic disease): individuals with weak disease symptoms;
  - $I_{ms}$ (medium symptoms): individuals with disease symptoms (e.g., fever or cough) who did not require hospitalization. The average sojourn time in states $I_{ps}$ and $I_{ms}$ are the same as in state *A*;
  - $I_{ss}$ (severe symptoms): individuals severely infected requiring hospitalization. They stayed in $I_{ss}$ before being hospitalized. The mean sojourn time in $I_{ss}$ was $t_{bh}$.
  Given the onset of symptoms, the probabilities of states $I_{ps}$, $I_{ms}$, and $I_{ss}$ are, respectively, $p_{I_{ps}}$, $p_{I_{ms}}$, and $p_{H}$ (probabilities summing to one).
  An individual leaving states *A*, $I_{ps}$ or $I_{ms}$ ends in state $R_{1}$ (removed individuals).
- When leaving $I_{ss}$, individuals enter the hospitalization period, which corresponds to four states:
  - $H_{1}$: individuals hospitalized before it was determined whether they needed intensive care or not;
  - $H_{2}$: individuals hospitalized without the need for ICU;
  - ICU: individuals hospitalized in ICU;
  - $H_{3}$: individuals hospitalized after leaving ICU.
  From hospital, all individuals end in one of the two absorbing states:
  - *D* (deceased at hospital);
  - $R_{2}$ (removed): individuals who recovered.
  During the hospitalization period, each individual follows a Markov chain dynamics (the dotted frame in Figure 4), with daily transition probabilities noted $p_{H2|H1}$, $p_{ICU|H1}$, $p_{H3|ICU}$, $p_{D|H2}$, $p_{R|H2}$, $p_{D|H3}$, and $p_{R|H3}$.

The age distribution of the population came from the INSEE (Institut National de la Statistique et des Études Économiques); nine age groups were considered: 8 ten-year age groups from 0 to 79 years, plus an extra age-group with individuals aged 80 or older. The contact matrix was available for France, on the basis of these age groups [18]. The number of daily contacts per individual was set at the beginning of the simulation and drawn from a Poisson distribution, with a parameter set to the corresponding element of the contact matrix. At each time step, the daily contacts between infectious and susceptible agents were drawn at random.

#### 4.1.2. Transition Probabilities

The disease spreads with infectious contacts that move individuals from state S to state E. Daily contacts between agents were based on a contact matrix (*C*), the element ($C_{i,j}$) of *C* being the mean number of individuals from age group *j* encountered per day by individuals from age group *i*. $C_{i.}=\sum_{j}C_{i,j}$ is the mean number of individuals encountered per day by individuals from age group *i*. The daily number of individuals encountered by a new agent was obtained from a Poisson distribution, with parameter $C_{i.}$, the expected proportions of contacts aged *j* being $C_{i,j}$/$C_{i.}$.

The probability ($P_{\mathrm{infect}}$) of an individual susceptible to infection because of a contact with an infectious individual was decomposed into a product of four terms:

(1) $$P_{\mathrm{infect}}\left(\mathrm{age},\mathrm{NPI},\mathrm{strain},Z\right)=\beta_{1,\mathrm{age}}\;\beta_{2,\mathrm{NPI}}\;\beta_{3,\mathrm{strain}}\;i_{Z}$$

where:

- $\beta_{1,\mathrm{age}}$ was the estimated baseline probability of infection, as a function of age (based on the age of the susceptible individual).
- $\beta_{2,\mathrm{NPI}}$, NPI∈{Moderate,Intensive,Extended}, estimated the effects of three NPI levels: moderate, intensive, and extended. The underlying hypotheses were that the NPI effects are independent from the state of the infectious individual, age group, or virus strain. Fixing $\beta_{2,\mathrm{Moderate}}=1$, parameters $\beta_{2,\mathrm{Intensive}}$, and $\beta_{2,\mathrm{Extended}}$ estimated the relative reductions in disease transmission under intensive- and extensive-NPIs, in comparison to moderate-NPIs. Parameter $\beta_{2,\mathrm{NPI}}$ was interpreted as a coefficient, reducing the mean number of daily contacts, mean duration of contacts, and/or their infectiousness ($0\leq \beta_{2,\mathrm{NPI}}\leq 1$).
- $\beta_{3,\mathrm{strain}}$, strain∈{historical,Alpha}, estimated the relative contagiousness of each virus strains, $\beta_{3,\mathrm{Alpha}}$, estimating the relative contagiousness of the Alpha variant, compared to that of the historical strain ($\beta_{3,\mathrm{historical}}=1$).
- $Z\in \{E,I_{p},A,I_{ps},I_{ms},I_{ss}\}$ is the state of the infectious individual, $i_{Z}$ being the relative infectiousness of individuals in state *Z*. It was fixed to 1 for $I_{ms}$ and $I_{ss}$ states (reference states), and to 0.55 for $I_{p}$, $I_{A}$, and $I_{ps}$ states, according to the literature (Table 7).

Sojourn times, in states (*E*, *A*, $I_{s}$, $I_{ps}$, $I_{ms}$, and $I_{ss}$), were generated from Weibull distributions. When more than one destination state was possible, the transition was selected at random using the corresponding probabilities (i.e., $p_{A}$, $p_{I_{ps}}$, $p_{I_{ms}}$, and $p_{I_{ss}}$).

For the right-hand part of the model (dotted frame, Figure 4), each day, for each individual in state $H_{1}$, $H_{2}$, $H_{3}$, or ICU, the state transition was drawn with probabilities $p_{H2|H1}$, $p_{ICU|H1}$, $p_{H3|ICU}$, $p_{D|H2}$, $p_{R|H2}$, $p_{D|H3}$, and $p_{R|H3}$, where staying in the same state (i.e., self-loops) were the complementary events.

The proportion of asymptomatic forms was set to 20% (Table 7). The relative infectiousness of prodromal, asymptomatic, and paucisymptomatic forms of the historical strain was set to $i_{I_{p}}=i_{A}=i_{I_{ps}}=0.55$; medium and severe symptoms forms were taken as references, with $i_{I_{ms}}=i_{I_{ss}}=1$ (Table 7). The proportions of various symptomatic forms, paucisymptomatic forms, medium symptoms, and severe symptoms were specified, depending on age (Table 6).

### 4.2. Scenarios

In agreement with the three levels of NPIs, three scenarios were defined, the first one being the use of intensive-NPIs and the second being the use of extended-NPIs. The third scenario, termed relaxed-NPIs, was built as an alternation of 45-day periods of intensive and moderate protective measures, the first period being one of intensive measures.

The expected effects of the three protective scenarios were then compared.

#### 4.2.1. Vaccination Effects

The expected effects of vaccination were set at their most recently estimated levels of efficacy in preventing severe forms of the disease; that is, 94% [5,6] and 75% [4], depending on the vaccine. When vaccination was effective, a vaccinated individual could develop only an asymptomatic form of the disease. Putative reductions of virus transmission were set to 50, 75, and 90%, the reduction of transmission probability being independent of the vaccine. An age-based priority for being vaccinated was used. Four vaccination priorities were considered and all individuals of a given age group had to receive their first injection before giving the first injections to the next priority age group. The age priorities considered were: 75 and older, 65 and older, 55 and older, and then the rest of the population. In the rest of the population, two vaccination options were compared: vaccination of individuals aged 10 years and over vs. vaccination at all ages (vaccination is currently under investigation in the youngest population). The effects of four vaccination campaigns, over 6, 9, 12, and 18 months, were compared. At the end of each vaccination campaign, all individuals eligible to vaccination should have received their second injection. In all simulations, vaccines with 94% efficacy were attributed to all individuals aged 75 or older and to all individuals aged 54 or younger. For individuals aged between 55 and 74, vaccines with 94% efficacy were attributed to 5/6 of individuals, whereas vaccines with 75% efficacy were attributed to the remaining 1/6 of individuals, aged between 55 and 74. These choices were made to fit vaccine availability for different age groups, as applied in France. An individual was considered to be fully vaccinated after receiving two injections of any vaccine with an interval of 42 days. In all simulations, vaccine supplies were considered sufficient to follow the study vaccination schedule. Furthermore, all vaccines were considered as efficient against the historical strain as against new variants, similar to the Alpha variant [19].

#### 4.2.2. Statistical Analyses

Due to the stochasticity of the agent-based simulation, 50 simulations were performed with each set of parameters. Each run involved 1,300,000 agents (i.e., 2% of the French metropolitan population—nearly 64.5 million inhabitants). To obtain estimations for France, the simulation results were multiplied by 50. The mean values and standard errors of the mean across the runs were plotted.

#### 4.2.3. Outcome Criteria

The outcome criteria were: the cumulative number of hospital deaths of COVID-19 patients, as well as the cumulative number of removed individuals due to the disease, from the beginning of the epidemic up to 30 June 2022, and the daily prevalence of COVID-19 patients in ICU beds and its saturation indicators during the simulation period (1 January 2021 to 30 June 2022).

The saturation of ICU beds was calculated as the cumulative number of cases requiring the ICU when all beds were already occupied. Three thresholds were considered for scenarios without vaccination: 5000 (the current number of ICU beds), 8000 (adding ICU beds in intermediate care units), and 12,000 (adding ICU beds in conventional care units).

To evaluate the occupation rate of the ICU under the various vaccination campaign scenarios, the numbers of days during which ICU occupancy exceeded the thresholds of 1000, 2000, and 3000 beds were calculated.

### 4.3. Model Calibration

The parameters of the model (Figure 4) obtained from the literature are shown in Table 7, and those estimated by calibration are shown in Table 6. The calibration was performed using public data from Santé Publique France (https://www.data.gouv.fr/fr/datasets/donnees-hospitalieres-relatives-a-lepidemie-de-covid-19/, accessed on 15 October 2020). Comparisons between those reference data and the simulation outputs, obtained from the estimated parameters, are shown in supplementary Figures S1–S5.

The left-hand part of the model was calibrated independently of the right-hand part (the dotted frame in Figure 4). For the left-hand part (‘disease spreading’), the parameters were estimated by likelihood maximization using daily hospital admissions from 1 September 2020 to 31 January 2021. The maximization was performed by the Nelder–Mead simplex method [20]. To reduce stochastic error, the calibration used daily hospital admissions averaged over 50 simulations. During each simulation, depending on the period, parameter $\beta_{2,\mathrm{NPI}}$ took three values, with NPI∈{Moderate,Intensive,Extended}. It was set to $\beta_{2,\mathrm{Moderate}}$ from 1 September 2020 to 29 October 2020 (before the second lockdown in France), then to $\beta_{2,\mathrm{Extended}}$ from 30 October 2020 to 30 November 2020 (second lockdown) and $\beta_{2,\mathrm{Intensive}}$ from 1 December 2020 to 31 January 2021.

Parameter $\beta_{2,\mathrm{Moderate}}$ was set to 1, as a reference, whereas $\beta_{2,\mathrm{Intensive}}$ and $\beta_{2,\mathrm{Extended}}$ were calibrated. The last parameter $\beta_{3,\mathrm{Alpha}}$ was estimated as the relative infectiousness of the Alpha variant, in comparison with the historical strain, for which $\beta_{3,\mathrm{historical}}$ was set to 1. Santé Publique France has estimated that, in France, on 8 January 2021, the Alpha variant was responsible for 3.3% of all new infections. In the simulations, during calibration, the presence of the Alpha variant was initiated on this date to 3.3%.

The right-hand part of the model (‘outcomes of hospitalized cases’) was calibrated by likelihood maximization, using the daily prevalence of *D* (deceased at hospital), ICU, $R_{2}$ (recovered after hospitalization), and daily prevalence of hospitalization (sum of $H_{1}$, $H_{2}$, and $H_{3}$). During calibration the input for this part of the model (i.e., the new cases arriving in state $H_{1}$) was the daily hospital admissions obtained from Santé Publique France, and, thus, did not require using the right-hand part of the model in these simulations. To handle parameter bounds (e.g., make all daily probabilities range between 0 and 1), the likelihood maximization relied on the L-BFGS-B method [21]. Due to the sensitivity to the daily hospital admissions used as input, all Santé Publique France data were smoothed using a sliding average over an interval of three days.

This right-hand part of the model could not be calibrated over a single period ranging from 1 September 2020 to 30 April 2021 (no satisfying convergence). This is likely to be due to changes in protocols and/or some probabilities (e.g., ICU stay or death), relative to the Alpha variant. Thus, it has been calibrated over two different periods. The first one ranged from 1 September to 31 December 2020, to grasp the effect of the historical strain (very small number of cases, related to new variants in France over that period). The second period was from 1 March to 30 April 2021, to capture the dynamics of the new variants (majority of cases of Alpha variant in France over that period). Then, in the different scenarios with the presence of a variant, the values of the parameters obtained for the first period were used in the simulation steps performed before 1 March 2021, while the second set of parameter values was used for the simulation steps from 1 March 2021. In scenarios where there was no variant, only the first set of parameter values was used in all simulation periods.

## 5. Conclusions

The main conclusion of this study is that the key point in the race against the historical COVID-19 strain and its variants is to optimise a combination of vaccination campaign and adequate NPIs. The proposed adaptive model allowed estimating the combined effect of vaccination campaign duration, virus contagiousness, and NPI level on the historical strain and Alpha variant. It appeared mandatory to vaccinate the majority of the French population within one year or less.

The flexibility of the model allows it to follow up the course of the current epidemic, taking into account the progress of the vaccination coverage and emergence of new variants, with the aim of adapting the NPIs until achieving herd immunity.

## Figures and Tables

**Table 1:** Historical strain without vaccination. Cumulative numbers of individuals removed and hospital deaths on 30 June 2022.

NPIs	Removed	Deceased
Relaxed-NPIs	26,397,570	372,973
Intensive-NPIs	16,688,896	202,411
Extended-NPIs	7,906,676	60,771

**Table 2:** Historical strain and Alpha variant development without vaccination. Cumulative numbers of individuals removed and hospital deaths on 30 June 2022.

NPIs	Removed	Deceased
Relaxed-NPIs	48,293,980	621,289
Intensive-NPIs	45,537,214	570,555
Extended-NPIs	20,551,828	213,715

**Table 3:** Results of a vaccine with 90% transmission reduction, with the historical strain and Alpha variant under different vaccination ages, campaign duration, and non-pharmaceutical interventions (NPIs). Cumulative numbers of individuals removed and deceased on 30 June 2022, cumulative ICU bed-days required, maximum daily ICU bed-days required, and intensive care unit (ICU) overload (exceeding bed–days capacity) over a period of 18 months.

NPIs	Vaccination Ages	Campaign Duration (Days)	Removed	Deceased	Cumulative ICU Bed-Days	Max Bed-Days /On Day	Days of ICU Overload If Max Available Beds Is		
							**1000**	**2000**	**3000**
Intensive-NPIs	10+	180	8,625,521	56,916	192,728	2926/30	69	48	0
Intensive-NPIs	10+	270	9,446,952	59,006	253,715	2928/30	86	57	0
Intensive-NPIs	10+	360	10,715,225	60,552	341,083	3001/27	109	69	1
Intensive-NPIs	10+	540	14,160,343	66,375	662,125	3281/80	270	155	72
Intensive-NPIs	All	180	8,537,674	56,466	180,477	2853/26	65	46	0
Intensive-NPIs	All	270	9,190,934	57,836	230,283	2855/30	79	52	0
Intensive-NPIs	All	360	10,074,304	58,819	304,086	2883/29	100	61	0
Intensive-NPIs	All	540	13,321,101	65,539	556,832	3250/36	256	107	27
Extended-NPIs	10+	180	7,681,105	53,277	101,474	2553/3	42	23	0
Extended-NPIs	10+	270	7,702,427	53,331	107,054	2461/7	44	22	0
Extended-NPIs	10+	360	7,725,029	54,139	118,005	2634/7	47	26	0
Extended-NPIs	10+	540	7,816,371	55,280	122,126	2578/4	47	25	0
Extended-NPIs	All	180	7,695,726	53,418	106,390	2592/3	44	24	0
Extended-NPIs	All	270	7,785,962	54,464	116,136	2633/7	47	26	0
Extended-NPIs	All	360	7,734,358	53,728	113,134	2615/7	45	23	0
Extended-NPIs	All	540	7,749,345	54,387	113,459	2517/7	46	22	0

**Table 4:** Results of a vaccine with 75% transmission reduction, with the historical strain and Alpha variant under different vaccination ages, campaign duration, and non-pharmaceutical interventions (NPIs). Cumulative numbers of individuals removed and deceased on 30 June 2022, cumulative ICU bed-days required, maximum daily ICU bed-days required, and intensive care unit (ICU) overload (exceeding bed–days capacity), over a period of 18 months.

NPIs	Vaccination Ages	Campaign Duration (Days)	Removed	Deceased	Cumulative ICU Bed-Days	Max Bed-Days /On Day	Days of ICU Overload If Max Available Beds Is		
							**1000**	**2000**	**3000**
Intensive-NPIs	10+	180	8,848,757	57,720	207,524	3087/25	73	51	10
Intensive-NPIs	10+	270	9,726,806	58,995	270,539	2958/30	94	61	0
Intensive-NPIs	10+	360	11,501,532	61,280	397,393	3088/32	154	81	13
Intensive-NPIs	10+	540	15,243,733	67,387	741,392	3378/84	278	210	76
Intensive-NPIs	All	180	8,574,155	55,863	182,187	2787/20	67	47	0
Intensive-NPIs	All	270	9,427,345	58,267	248,697	2950/28	86	57	0
Intensive-NPIs	All	360	10,826,562	60,202	360,763	3061/31	123	73	12
Intensive-NPIs	All	540	14,114,090	65,830	629,200	3090/37	265	137	24
Extended-NPIs	10+	180	7,726,318	53,926	106,497	2544/8	43	24	0
Extended-NPIs	10+	270	7,799,063	54,892	114,168	2553/7	45	25	0
Extended-NPIs	10+	360	7,689,234	53,274	109,427	2497/8	44	21	0
Extended-NPIs	10+	540	7,782,863	54,260	122,188	2565/7	48	24	0
Extended-NPIs	All	180	7,691,260	52,927	103,575	2496/8	43	23	0
Extended-NPIs	All	270	7,801,188	54,828	113,826	2726/7	45	25	0
Extended-NPIs	All	360	7,760,065	54,589	115,912	2535/7	47	25	0
Extended-NPIs	All	540	7,857,610	56,095	127,551	2746/8	49	27	0

**Table 5:** Results of a vaccine with 50% transmission reduction, with the historical strain and Alpha variant under different vaccination ages, campaign duration, and non-pharmaceutical interventions (NPIs). Cumulative numbers of individuals removed and deceased on 30 June 2022, cumulative ICU bed-days required, maximum daily ICU bed-days required, and intensive care unit (ICU) overload (exceeding bed–days capacity) over a period of 18 months.

NPIs	Vaccination Ages	Campaign Duration (Days)	Removed	Deceased	Cumulative ICU Bed-Days	Max Bed-Days /On Day	Days of ICU Overload If Max Available Beds Is		
							**1000**	**2000**	**3000**
Intensive-NPIs	10+	180	9,202,882	57,504	216,751	2870/17	80	52	0
Intensive-NPIs	10+	270	11,043,587	59,056	336,887	2926/31	131	72	0
Intensive-NPIs	10+	360	14,036,762	63,840	556,218	3031/38	241	119	11
Intensive-NPIs	10+	540	19,133,475	74,014	1,106,397	5219/134	298	258	207
Intensive-NPIs	All	180	9,023,689	56,264	202,522	2830/25	75	49	0
Intensive-NPIs	All	270	10,527,221	59,584	310,561	3071/28	113	62	10
Intensive-NPIs	All	360	13,036,072	62,685	481,481	3117/27	218	96	22
Intensive-NPIs	All	540	17,927,080	70,458	926,777	3877/145	291	249	181
Extended-NPIs	10+	180	7,771,015	54,196	107,426	2530/2	44	24	0
Extended-NPIs	10+	270	7,772,026	54,103	111,120	2502/8	45	23	0
Extended-NPIs	10+	360	7,782,238	54,307	117,712	2541/9	46	22	0
Extended-NPIs	10+	540	7,775,685	54,302	122,420	2486/8	48	23	0
Extended-NPIs	All	180	7,705,102	53,347	106,052	2571/7	43	23	0
Extended-NPIs	All	270	7,778,149	54,255	110,905	2553/7	46	22	0
Extended-NPIs	All	360	7,784,818	54,110	120,321	2593/6	48	24	0
Extended-NPIs	All	540	7,836,271	55,120	128,861	2655/5	48	26	0

**Table 6:** Estimated parameters (the values in italics for the left-hand part of the model are extracted from a previous work [15]).

Age	0–9	10–19	20–29	30–39	40–49	50–59	60–69	70–79	80+
Left-hand part of the model									
$\beta_{1,\mathrm{age}}$	0.01	0.01	0.017	0.008	0.007	0.012	0.016	0.16	0.164
$p_{I_{ps}}$	0.249	*0.249*	*0.247*	0.243	0.242	*0.225*	*0.209*	*0.189*	0.031
$p_{I_{ms}}$	0.746	*0.748*	*0.741*	0.730	0.727	*0.674*	*0.626*	*0.568*	0.092
$p_{I_{ss}}$	0.006	*0.003*	*0.012*	0.026	0.031	*0.102*	*0.166*	*0.243*	0.877
$\beta_{2,\mathrm{Intensive}}$	0.783 for all ages								
$\beta_{2,\mathrm{Extended}}$	0.534 for all ages								
$\beta_{3,\mathrm{Alpha}}$	1.572 for all ages								
Right-hand part of the model for historical strain (calibration period September 2020–December 2020)									
$p_{ICU|H1}$	0.151	0.5	0.077	0.078	0.117	0.146	0.228	0.227	0.128
$p_{H2|H1}$	0.849	0.95	0.923	0.922	0.874	0.854	0.771	0.773	0.872
$p_{H3|ICU}$	0.149	0.055	0.143	0.065	0.058	0.049	0.051	0.062	0.22
$p_{D|H2}$	0	0	0	0.001	0.001	0.002	0.006	0.012	0.019
$p_{R|H2}$	0.526	0.462	0.442	0.265	0.188	0.142	0.098	0.07	0.036
$p_{D|H3}$	$3\times 10^{-4}$	$5\times 10^{-4}$	0.001	0.002	0.005	0.007	0.009	0.009	0.008
$p_{R|H3}$	0.039	0.001	0.018	0.016	0.016	0.01	0.01	0.009	0.067
Right-hand part of the model for Alpha variant (calibration period March 2021–April 2021)									
$p_{ICU|H1}$	0.052	0.031	0.077	0.071	0.116	0.157	0.280	0.268	0.098
$p_{H2|H1}$	0.948	0.967	0.902	0.918	0.873	0.841	0.396	0.732	0.895
$p_{H3|ICU}$	0.079	0.033	0.070	0.033	0.033	0.033	0.075	0.057	0.139
$p_{D|H2}$	0	0	0.001	0.001	0.002	0.003	0.009	0.012	0.016
$p_{R|H2}$	0.288	0.193	0.165	0.151	0.115	0.082	0.040	0.035	0.030
$p_{D|H3}$	0	0	0.001	0.005	0.004	0.007	0.001	0.004	0.014
$p_{R|H3}$	0.084	0.052	0.211	0.057	0.055	0.053	0.102	0.059	0.073

**Table 7:** Parameters sojourn time, relative infectiousness, and rate of asymptomatic subjects, as obtained from the literature.

Parameter	Sources	Value
Sojourn time		
$t_{i}$	see [15]	5.1
$t_{p}$	see [15]	1.5
$t_{s}$	see [15]	7
$t_{bh}$	see [15]	3
Relative infectiousness		
$i_{Ims}$	[16]	1
$i_{Iss}$	[16]	1
$i_{A}$	[16]	0.55
$i_{Ip}$	[16]	0.55
$i_{Ips}$	[16]	0.55
Rate of asymptomatic subjects		
$p_{A}$	[14,17]	0.20
Proportion new variant (8 January 2021)		
Alpha variant	Santé Publique France	3.3%

## Data Availability

All data generated during this study are available from the corresponding author on reasonable request. The prevalence values of Alpha variant in France are publicly available from Santé Publique France (https://www.santepubliquefrance.fr/ (accessed on 15 October 2020)). The data used for estimation are also publicly available from Santé Publique France (https://www.data.gouv.fr/fr/datasets/donnees-hospitalieres-relatives-a-lepidemie-de-covid-19/ (accessed on 15 October 2020)). Prevalence of Removed individuals in France in 1 January 2021, are available from the publicly available data of Institut Pasteur (https://modelisation-covid19.pasteur.fr/realtime-analysis/infected-population/ (accessed on 15 October 2020)). The source code is available on gitlab: https://gitlab.com/Alnindra/covdyn_abm/ (accessed on 15 October 2020).

## Supplementary Materials

The following are available at https://www.mdpi.com/article/10.3390/vaccines9121462/s1. Figure S1: The calibrated hospital admission from 1 September 2020 to 31 January 2021. Figure S2: The calibrated prevalence of *H* and ICU from 1 September 2020 to 31 December 2020. Figure S3: The calibrated prevalence of $R_{2}$ and *D* from 1 September 2020 to 31 December 2020. Figure S4: The calibrated prevalence of *H* and ICU from 1 March 2021 to 30 April 2021. Figure S5: The calibrated prevalence of $R_{2}$ and *D* from 1 March 2021 to 30 April 2021.

## Appendix A. Members of the CovDyn (Covid Dynamics) Group, by Main Affiliation

LBBE–HCL

Mathieu Fauvernier, François Gueyffier, Jean Iwaz, Delphine Maucort-Boulch, Simon Pageaud, Muriel Rabilloud, Pascal Roy.

LSAF

Alexis Bienvenüe, Anne Eyraud-Loisel, Romain Gauchon, Pierre-Olivier Goffard, Nicolas Leboisne, Stéphane Loisel, Xavier Milhaud, Denys Pommeret, Yahia Salhi.

LAMA

Pierre Vandekerkhove.

CIRI-HCL

Philippe Vanhems.

LMFA

Jean-Pierre Bertoglio.

LTDS

Nicolas Ponthus.

INL

Christelle Yeromonahos.

LIRIS

Stéphane Derrode, Véronique Deslandres, Mohand-Saïd Hacid, Salima Hassas, Catherine Pothier, Christophe Rigotti.

ICJ

Thibault Espinasse, Samuel Bernard, Philippe Michel, Vitaly Volpert.

INRIA

Mostafa Adimy.

CHU de Rouen Unité INSERM 1018, CESP

Jacques Benichou.

CHU de Rouen LIMICS INSERM U1142, Université de Rouen/Sorbonne Université

Stéfan Darmoni.

CHU de NICE, Université de Nice

Pascal Staccini.
