# Supplementary material for: Expected Evolution of COVID-19 Epidemic in France for Several Combinations of Vaccination Strategies and Barrier Measures
Source: Vaccines (Basel). 2021 Dec 10;9(12):1462. doi: 10.3390/vaccines9121462 (PMC8708137; doi:10.3390/vaccines9121462)
Supplement: Supplementary file 1 [file vaccines-09-01462-s001.zip › vaccines-1450256 supplementary.pdf]

vaccines

MDPI

Expected Evolution of COVID-19 Epidemic in France for Several Combinations of Vaccination Strategies and Barrier Measures

Simon Pageaud [1,2,3,4,5,6], Catherine Pothier [7,8], Christophe Rigotti [7,8,9] and Anne Eyraud-Loisel [5], Jean-Pierre Bertoglio [10,11], Alexis Bienvenüe [5], Nicolas Leboisne [5], Nicolas Ponthus [11,12,13], Romain Gauchon [5], François Gueyffier [1,2,3,4], Philippe Vanhems [14,15], Jean Iwaz [1,2,3,4], Stéphane Loisel [5], Pascal Roy [1,2,3,4,12,*] and on behalf of the Group CovDyn (Covid Dynamics)

1. Université de Lyon, F-69000, Lyon, France; simon.pageaud@gmail.com (S.P.); francois.gueyffier@univ-lyon1.fr (F.G.); jean.iwaz@chu-lyon.fr (J.I.)
2. Université Claude Bernard Lyon 1, F-69100, Villeurbanne, France
3. CNRS UMR 5558, Laboratoire de Biométrie et Biologie Évolutive, F-69100, Villeurbanne, France
4. Service de Biostatistique-Bioinformatique, Pôle Santé Publique, Hospices Civils de Lyon, F-69003, Lyon, France
5. Laboratoire de Sciences Actuarielle et Financière (LSAF), Institut de Science Financière et d'Assurances (ISFA), Université Claude Bernard Lyon 1, F-69007, Lyon, France; anne.loisel@univ-lyon1.fr (A.E.-L.); alexis.bienvenue@univ-lyon1.fr (A.B.); nicolas.leboisne@univ-lyon1.fr (N.L.); romain.gauchon@laposte.net (R.G.); stephane.loisel@univ-lyon1.fr (S.L.)
6. Fondation du Risque, Groupe Louis Bachelier, F-75002, Paris, France
7. CNRS UMR 5205, Laboratoire d'InfoRmatique en Image et Systèmes d'Information (LIRIS), F-69621, Villeurbanne, France; catherine.pothier@insa-lyon.fr (C.P.); christophe.rigotti@insa-lyon.fr (C.R.)
8. Institut National des Sciences Appliquées de Lyon (INSA), F-69621, Villeurbanne, France
9. INRIA Grenoble-Rhône-Alpes, F-38334, Montbonnot, France
10. CNRS UMR 5509, Laboratoire de Mécanique des Fluides et d'Acoustique (LMFA), F-69130, Ecully, France; jean-pierre.bertoglio@ec-lyon.fr
11. École Centrale de Lyon, F-69130, Lyon, France; nicolas.ponthus@gmx.fr
12. CNRS UMR 5513, Laboratoire de Tribologie et Dynamique des Systèmes (LTDS), F-69130, Écully, France
13. École Nationale des Travaux Publics de l'État (ENTPE), F-69120, Vaulx-en-Velin, France
14. Service d'Hygiène, Épidémiologie, Infectiovigilance et Prévention, Hôpital Edouard Herriot, Hospices Civils de Lyon, F-69003, Lyon, France; philippe.vanhems@chu-lyon.fr
15. Centre International de Recherche en Infectiologie (CIRI: Inserm U1111, CNRS UMR 5308, École Nationale Supérieure de Lyon), F-69007, Lyon, France

* Correspondence: Correspondence: pascal.roy@chu-lyon.fr;

Citation: Pageaud, S.; Pothier, C.; Rigotti, C.; Eyraud-Loisel, A.; Bertoglio, J.-P.; Bienvenüe, A.; Leboisne, N.; Ponthus, N.; Gauchon, R.; Gueyffier, F.; et al. Expected Evolution of COVID-19 Epidemic in France for Several Combinations of Vaccination Strategies and Barrier Measures. Vaccines 2021, 9, 1462. https://doi.org/10.3390/vaccines9121462

Academic Editor: S. Louise Cosby

Received: 20 October 2021
Accepted: 2 December 2021
Published: 10 December 2021

Publisher's Note: MDPI stays neutral with regard to jurisdictional claims in published maps and institutional affiliations.

Copyright: © 2021 by the authors. Submitted for possible open access publication under the terms and conditions of the Creative Commons Attribution (CC BY) license (https://creativecommons.org/licenses/by/4.0/).

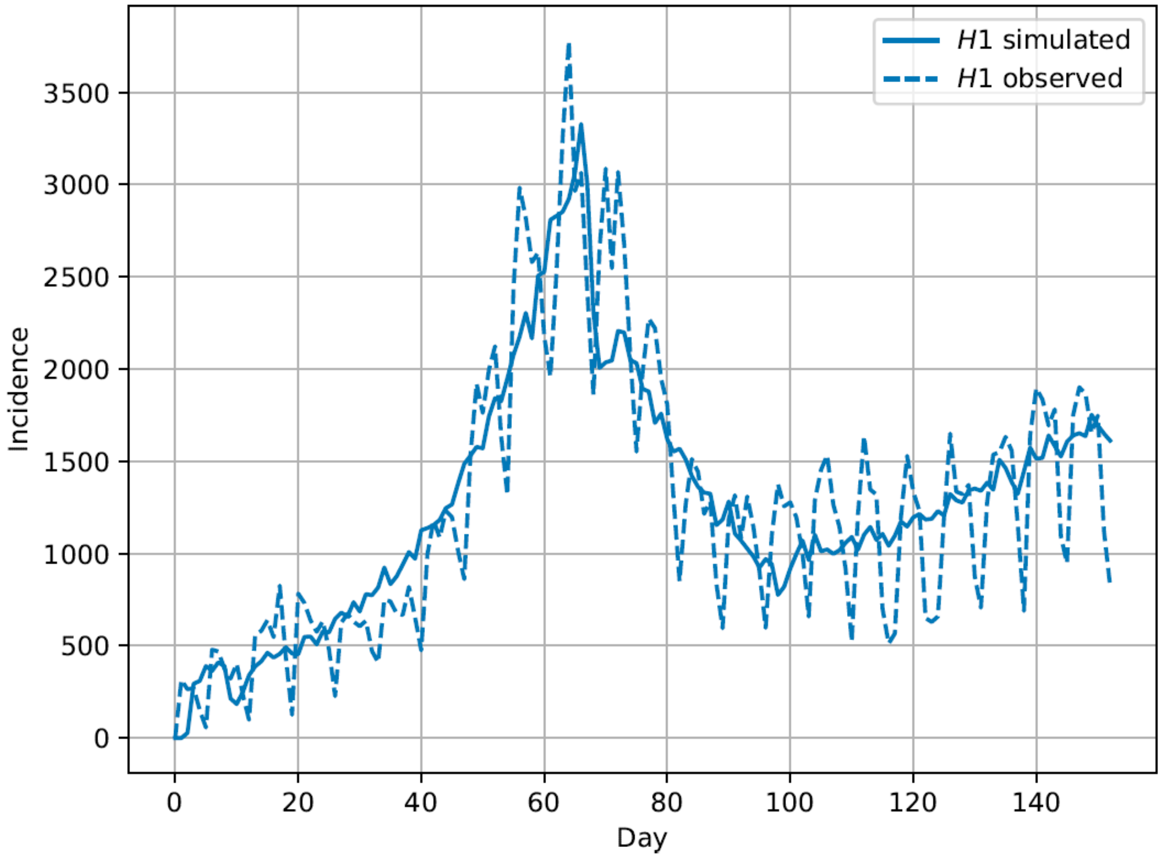

Figure S1. Calibration: observed and predicted values of daily hospital admissions (H1) from September 1, 2020 to January 31, 2021.

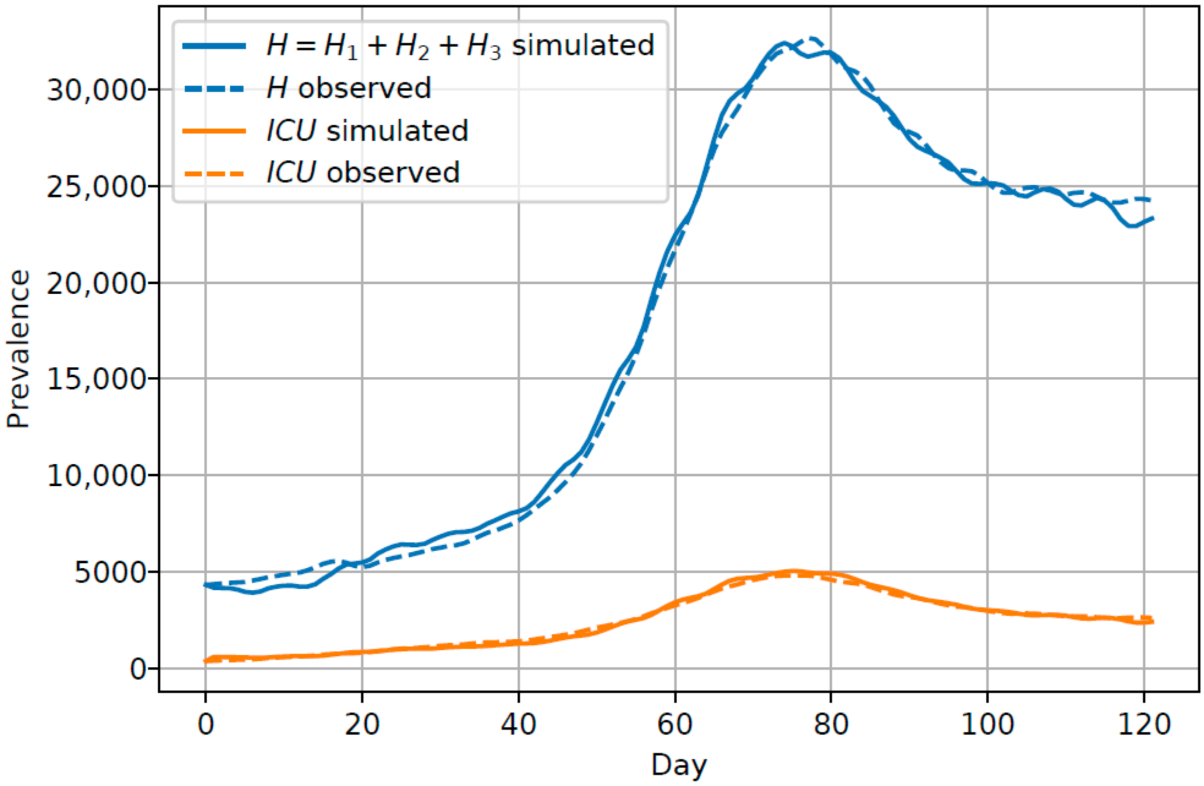

Figure S2. Calibration: observed and predicted prevalences for H and ICU from September 1, 2020 to December 31, 2020.

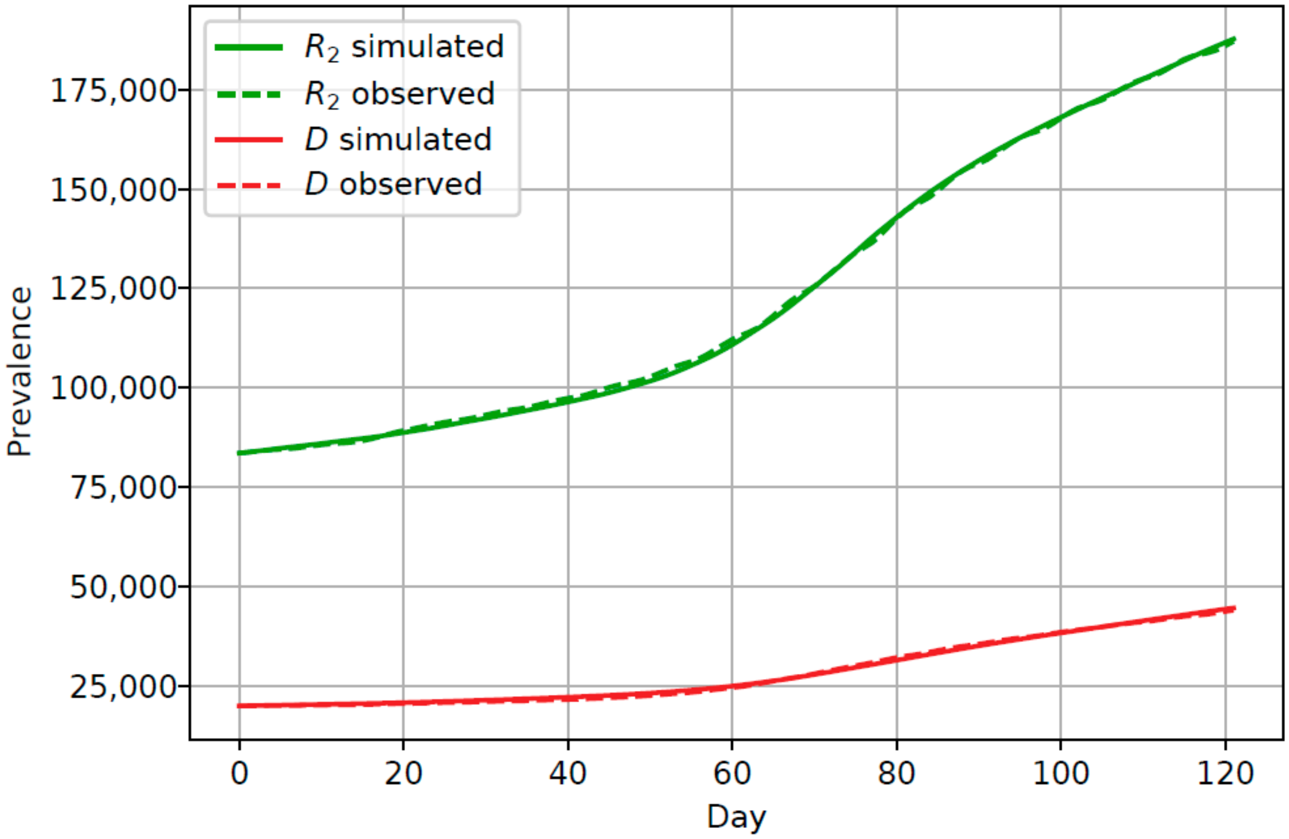

Figure S3. Calibration: observed and predicted prevalences for R2 and D from September 1, 2020 to December 31, 2020.

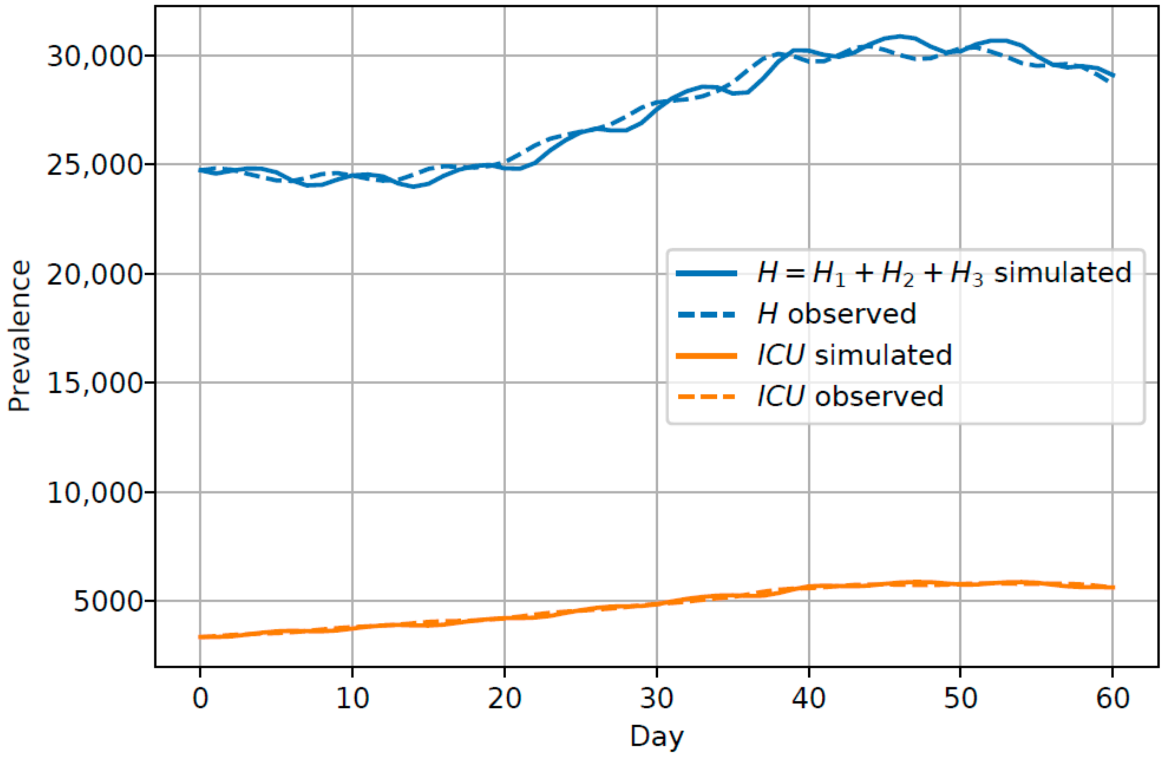

Figure S4. Calibration: observed and predicted prevalences for H and ICU from March 1, 2021 to April 30, 2021.

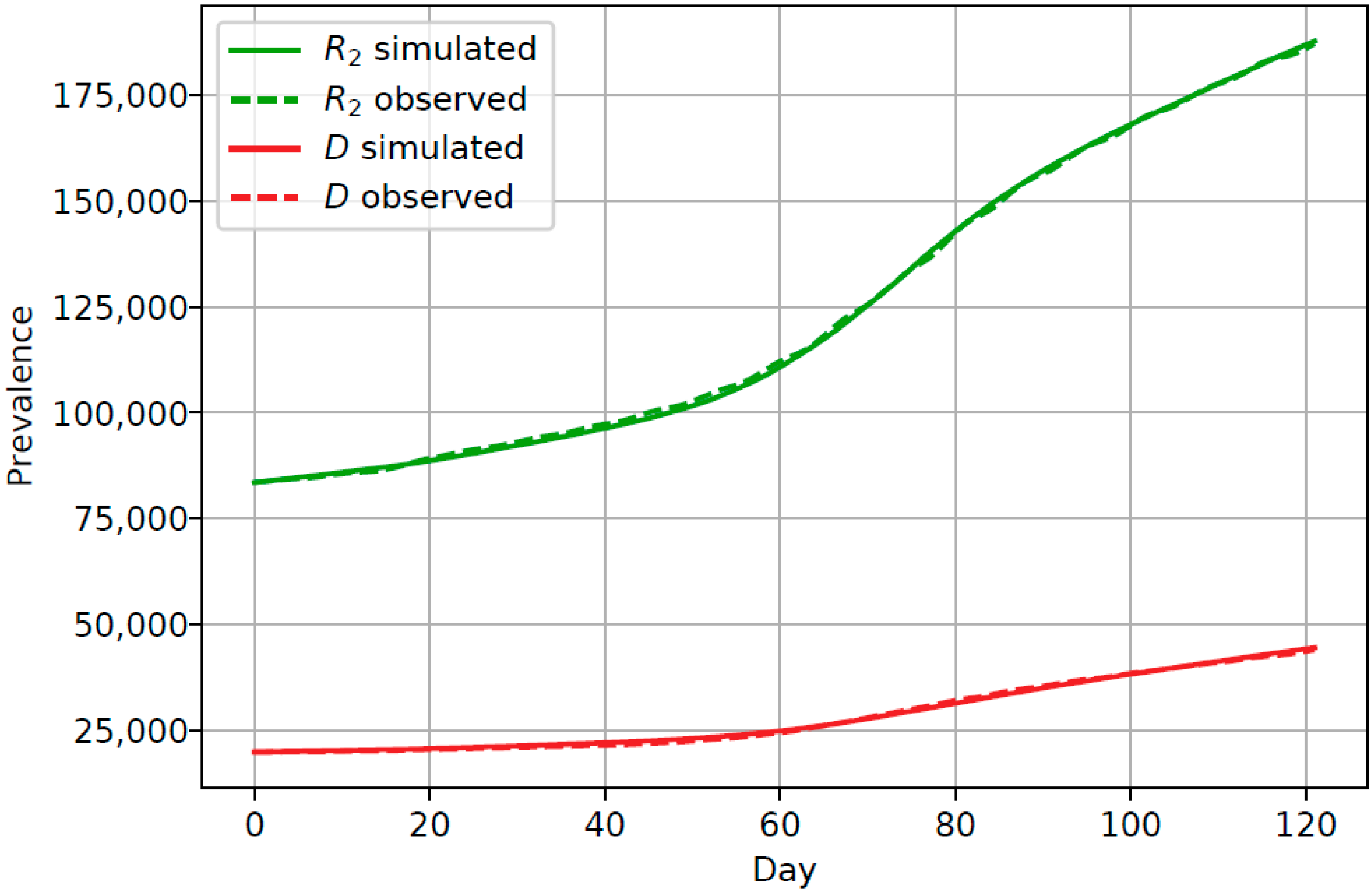

Figure S5. Calibration: observed and predicted prevalences for R2 and D from March 1, 2021 to April 30, 2021
